# Obesity, Diabetes and Atrial Fibrillation; Epidemiology, Mechanisms and Interventions

**DOI:** 10.2174/157340312803760749

**Published:** 2012-11

**Authors:** O Asghar, U Alam, SA Hayat, R Aghamohammadzadeh, AM Heagerty, RA Malik

**Affiliations:** 1 Division of Cardiovascular Sciences, The University of Manchester, UK; 2 Department of Cardiology, Imperial College Healthcare NHS Trust, London, UK

**Keywords:** Obesity, metabolic syndrome, epidemiology, atrial fibrillation, morbidity, mortality.

## Abstract

The last few decades have witnessed a global rise in adult obesity of epidemic proportions. The potential impact of this is emphasized when one considers that body mass index (BMI) is a powerful predictor of death, type 2 diabetes (T2DM) and cardiovascular (CV) morbidity and mortality [1, 2]. Similarly we have witnessed a parallel rise in the incidence of atrial fibrillation (AF), the commonest sustained cardiac arrhythmia, which is also a significant cause of cardiovascular morbidity and mortality. Part of this increase is attributable to advances in the treatment of coronary heart disease (CHD) and heart failure (HF) improving life expectancy and consequently the prevalence of AF. However, epidemiological studies have demonstrated an independent association between obesity and AF, possibly reflecting common pathophysiology and risk factors for both conditions. Indeed, weight gain and obesity are associated with structural and functional changes of the cardiovascular system including left atrial and ventricular remodeling, haemodynamic alterations, autonomic dysfunction, and diastolic dysfunction. Moreover, diabetic cardiomyopathy is characterized by an adverse structural and functional cardiac phenotype which may predispose to the development of AF [3]. In this review, we discuss the pathophysiological and mechanistic relationships between obesity, diabetes and AF, and the challenges posed in the management of this high-risk group of individuals.

## OBESITY, METABOLIC SYNDROME AND AF RISK

The link between obesity and AF was first recognized in retrospective analyses of incident AF in peri-operative cardiac surgical patients [4-7]. These early observations were subsequently supported by data from several large cohort studies. The first prospective study of incident AF was undertaken in subjects from the Framingham cohort, in which there was a 4-5% increase in AF risk for every unit increase in BMI, over a mean duration of 13.7 years [8]. Furthermore, this association remained unchanged even after adjusting for myocardial infarction (MI), hypertension (HT) and diabetes (DM), and increased across the range of obesity. In the same study, obesity was also associated with an increase in left atrial diameter, a recognized precursor of AF. However, the relationship between BMI and AF was lost following adjustment for left atrial size, suggesting AF risk in obese subjects is mediated through left atrial enlargement, a finding which has also been reported in subjects with the metabolic syndrome (MetS) [9, 10]. Indeed, left atrial enlargement is present in a significant proportion of obese adolescents and adults and is attenuated following weight reduction [11-14]. In the MONICA/KORA study, age, obesity and HT were independently associated with left atrial enlargement over a ten year period; obesity being the most powerful predictor (OR: 2.4 vs. 2.2; p<0.001) and the combination of obesity and HT demonstrated the greatest LA enlargement [15]. Other large prospective studies have reported comparable AF risk, for example in the Danish Diet, Cancer and Health study of 47,589 individuals followed up over a mean duration of 5.7 years, the incidence of AF/flutter was 1.2%, two thirds of which occurred in males [16]. The adjusted hazard ratios (HR) for AF/flutter were 2.35 (95% CI 1.70-3.25) and 1.99 (95% CI 1.31-3.02) in obese men and women respectively. In a recent meta-analysis, obesity was associated with a 49% increased risk of AF (RR 1.49, 95% CI 1.36-1.64) in 6 population studies, but no association was reported in post cardiac surgery studies (RR 1.02, 95% CI 0.99-1.06) [17]. Similarly, in the long term follow up of the Atherosclerosis Risk in Communities (ARIC) study cohort, 17.9% of incident AF was attributed to obesity (18-21). Data from long term follow up in Swedish men indicated an association between the risk of AF and BMI and body surface area in youth [18] and obesity was also associated with an increased risk of progression from paroxysmal AF to permanent AF [19]. In the Women’s Health Study of over 34,000 female healthcare professionals free of CVD, the investigators reported a linear relationship between BMI and AF characterized by a 4.7% (95% CI: 3.4-6.1% p<.0001) increased risk of AF for every 1 unit increase in BMI [20]. This association was maintained whichever classification of obesity was used and after adjusting for confounding variables and inflammatory markers. Importantly, a change in weight category during the first 5 years of follow up was associated with a corresponding change in AF risk. This risk was marginally greater in subjects who progressed from the non-obese (BMI ≤ 30) to obese (BMI ≥30) weight category than those who were obese at baseline and follow up (RR 1.41 vs. 1.32). Weight reduction from obese to non-obese was associated with a risk reduction to that of non-obese subjects at baseline. These findings would imply that AF risk in obese individuals is reversible, achievable through interventions such as weight reduction. Furthermore, the lower risk observed at follow up in the group who were obese at baseline supports the concept of an *obesity paradox *which has been reported in previous studies such as the the Atrial Fibrillation Follow-Up Investigation of Rhythm Management (AFFIRM) trial in which obese patients with AF had favourable outcomes compared with their non-obese counterparts [21, 22]. Clearly, further studies are required to investigate this phenomenon.

Although there is considerable overlap with obesity, Met S is generally considered to be a constellation of risk factors specific to the development of atherosclerotic cardiovascular disease and T2DM and is characterized by abdominal obesity, dyslipidaemia, HT and glucose intolerance [23]. Few robust studies have been carried out in MetS cohorts to assess AF risk. In the Niigata study, subjects with MetS had an increased risk of AF which was independently associated with age and obesity, irrespective of the definition of MetS used (AHA/NHLBI vs. NCEP-ATPIII) [24]. In multivariate models for individual components of the MetS, BMI and HT contributed the most to AF risk and interestingly, low HDL cholesterol was also strongly associated. In addition, AF risk was positively correlated with the number of MetS components exhibited, further supporting the notion that MetS simply represents a clustering of risk factors [25]. In a recent study of the Framingham Offspring cohort, the relationship between insulin resistance (IR) and incident AF in non-diabetic subjects was investigated over ten years of follow up [26]. Both adjusted (HR 1.27, 95% CI 0.92 to 1.76, p=0.15) and unadjusted (HR 1.10, 95% CI 0.83 to 1.45, p=0.52) analyses failed to demonstrate an association between IR and AF.

Although the mechanistic basis for the relationship between obesity, LA enlargement and AF is not completely understood, it is likely to be multifactorial involving haemodynamic disturbances, autonomic dysfunction and induction of the renin-aldosterone-angiotensin-system (RAAS), resulting in mechanical and electrical remodeling of the left atrium.

Obstructive sleep apnoea (OSA) is an independent risk factor for stroke and death [27]. Obesity is causally related to OSA and both conditions share common pathophysiological mechanisms. It is estimated that OSA is present in 40% of obese subjects and 20% of non-obese but overweight subjects, respectively [28, 29]. OSA increases the risk of AF following coronary bypass surgery, electrical cardioversion and catheter ablation [30, 31], and may directly promote the initiation and/or maintenance of AF through alterations in the electrophysiological properties of the cardiomyocytes [32]. This may be mediated *via* several different mechanisms including repetitive and prolonged hypoxemia, exaggerated intra-thoracic pressure oscillations with increased cardiac wall stress, systemic inflammation and diastolic dysfunction [33-38]. Cardiac autonomic dysfunction, another feature of OSA, may also play an important role in the pathophysiology of AF [39] and experimental work in animal models of OSA has demonstrated both the role of autonomic dysfunction in the induction of AF and the utility of vagal ablation for the prevention of AF [40, 41].

An early study by Gami *et al* established the strong association between OSA and AF [42]. In a later retrospective cohort study, they investigated the risk of incident AF in a cohort of 3,542 subjects referred for sleep studies over a mean follow up period up of 4.7 years [42, 43]. OSA and obesity were independently associated with incident AF in subjects under 65 years. These findings are important for two reasons; first the severity of nocturnal oxygen desaturation was positively correlated with AF risk and second, the association between obesity and AF risk remained after controlling for OSA. The explanation for the lower AF risk observed in the over 65 age groups in this study was unclear. However the results were consistent with previous studies and require further study.

## DIABETES AND AF RISK

Several studies have reported an association between DM and AF [44-47]. In the ARIC study, the incidence of AF in diabetic subjects was double that of non-diabetic subjects [48]. Furthermore, DM but not pre-diabetes was associated with an increased AF risk (HR 1.35, 95% CI 1.14-1.60) which was independently associated with fasting glucose and HbA1c. AF was more prevalent in subjects with pre-diabetes compared to controls and correlated positively with HbA1c [49]. In a recent meta-analysis, DM was associated with an increased AF risk (RR 1.39, 95% CI 1.10-1.75, p <0.001) which remained significant after correction for publication bias and multiple risk factors (RR 1.24, 95% CI 1.06-1.44, vs. 1.70, 1.29-2.22, p = 0.053) [50].

## ADIPOCYTOKINES – THE LINK BETWEEN FAT, INFLAMMATION AND AF

C-reactive protein (CRP) levels are increased in patients with persistent AF compared to controls and subjects with paroxysmal AF (PAF), thus suggesting a possible link between inflammation and AF [51-53]. A building literature supports the role of adipose tissue mediated inflammation and the development of AF. Recent work on visceral adipose tissue has concentrated on the role of epicardial fat in the pathogenesis of AF. Epicardial fat thickness correlates strongly with visceral fat on magnetic resonance imaging [54-56]. Individuals with permanent AF have a greater volume of epicardial fat compared with individuals with PAF, and those with either PAF or permanent AF have a greater volume of pericardial fat compared with controls [57]. This difference is predominantly related to atrial adiposity as opposed to periventricular epicardial fat thickness, which is comparable between all groups [58]. More specifically, adipose tissue thickness in the inter-atrial septum is positively correlated with BMI and left atrial volume and inversely correlated with plasma adiponectin levels [58]. Epicardial fat thickness is associated with an increased risk of AF whereas pericardial fat is predictive of LA volume in addition to AF prevalence, severity and poorer outcomes following catheter ablation [59, 60].

The effects of epicardial fat on the heart are mediated through a group of cytokines produced by adipocytes known as adipocytokines which possess inflammatory, anti-inflammatory and vasoactive properties implicated in the pathogenesis of several cardiovascular diseases [56, 61]. Several adipocytokines have been associated with AF including adiponectin, resistin, a pro-inflammatory cytokine associated with insulin resistance, and more recently, YKL-40, a general marker of inflammation [62-64]. Adiponectin levels are reduced in obesity and T2DM and increase following weight loss [65, 66]. Ybarra *et al* studied the relationship between LA size and adiponectin in obese subjects [67]. Adiponectin levels significantly correlated with indices of glycaemia, insulin and lipids and were significantly lower in both obese subjects and in obese subjects with an enlarged LA. The association with LA size persisted after adjustment for the homeostasis model assessment of insulin resistance (HOMA-IR), age, sex, and LV mass. Few studies have investigated the relationship between adipocytokines and AF. In a study of the Framingham offspring cohort, plasma resistin concentration was significantly associated with incident AF (HR 1.17, 95% CI 1.02-1.34, p=0.028), however this relationship was lost after adjustment for CRP [68] and adiponectin concentration did not predict incident AF. In contrast, adiponectin appears to have a protective effect following cardiac surgery [69, 70]. YKL-40 is associated with AF recurrence following catheter ablation, but did not predict successful cardioversion in patients with AF [71, 72]. In a Japanese study, adiponectin levels were elevated in patients with persistent AF compared to those with paroxysmal AF and controls [73]. The significance of such findings is unclear and further studies are required to determine the relationship between adipocytokines and AF.

The association between inflammation and AF has also been established *via* mechanistic *in vitro* studies [52, 74]. Inflammatory changes have been observed in atrial tissue from patients with lone AF [75] and at the cellular level, NAD(P)H oxidase activity and nitric oxide synthase uncoupling in the myocardium have been proposed as sources of free radicals contributing to oxidative stress in the atrial myocardium of patients with AF [76]. Whole-cell patch clamp studies of pulmonary vein cardiomyocytes have demonstrated that TNF-α incubation leads to altered calcium homeostasis and enhanced arrhythmogenicity [77].

## AUTONOMIC DYSFUNCTION AND THE ATRIAL SUBSTRATE

Cardiac autonomic neuropathy (CAN) the commonest manifestation of diabetic autonomic neuropathy (DAN), may be present during the pre-diabetic phase, and is a strong independent risk factor for morbidity and mortality [78-80]. This risk may be attributable to features of advanced CAN including sudden cardiac death (SCD), cardiac arrhythmias, silent myocardial ischaemia and lack of hypoglycaemia awareness [81]. Hypoglycaemia has been associated with an increased mortality risk which may be attributable to the proarrhythmic effects of hypoglycaemia on the QT interval [82]. However, despite the increased incidence of hypoglycaemia and mortality in the Action to Control Cardiovascular Risk in Diabetes (ACCORD) trial, there were fewer arrhythmia related deaths both during intensive treatment and following termination of this arm of the study [83]. AF risk is increased in diabetic subjects and this risk increases with diabetes duration and worsening glycaemic control [84, 85]. Although the exact role of glycaemia in the progression of CAN is not fully understood, several large clinical trials have demonstrated the beneficial effects of glycaemic control and multifactorial cardiovascular intervention on delaying the onset of CAN [86-89]. CAN is also associated with coronary microvascular dysfunction and diastolic dysfunction in diabetic subjects [90-92].

The cardiac autonomic nerves play a key role in the regulation of heart rate and an imbalance in autonomic tone can give rise to electrophysiological effects which may predispose to the development of AF [93]. The sino-atrial (SA) node and atrio-ventricular (AV) node are both influenced by autonomic tone, and vagal stimulation can result in marked changes in cardiac electrophysiology, including heterogeneous effects on the atrial refractory period (ARP), pacemaker activity and AV conduction [94, 95]. Increased vagal tone also contributes to the genesis of so called ‘vagal AF’, a phenomenon which is thought to play a key role in a subset of patients in whom primarily vagal abnormalities contribute to AF [96,97].

Sympathetic overactivity on the other hand, may precipitate AF and occurs following the induction of the RAAS. Increased levels of angiotensin II and decreased levels of the neuropeptide, substance P, are associated with post-operative AF incidence in patients undergoing CABG, thus implicating the role of sympathetic overactivity and reduced parasympathetic activity, respectively in the pathogenesis of AF [98]. Despite the early promise for the use of RAAS inhibitors in the prevention of AF, subsequent trials and meta-analyses have shown no benefit [99]. Another important manifestation of RAAS activation is myocardial fibrosis, which is mediated through pro-fibrotic agents such as angiotensin II and results in both electrical and mechanical remodeling of the left atrium predisposing to AF [100], [101].

Neural remodeling is thought to play a crucial role in increased AF vulnerability in DM and experimental models have shown alterations in sympathetic nerves in the right atrium with AF being enhanced by adrenergic activation [102,103]. Aberrant neurotransmission seems to be a key feature of arrhythmogenicity and a recently published study showed alterations in purinergic neurotransmission in the atria of diabetic rats [104]. AF may also be provoked in individuals with PAF and normal autonomic function by isoprenaline implying an increased susceptibility despite normal cardiovascular reflexes [105]. In addition, symptoms of dizziness at the onset of paroxysmal AF may be predicted by impaired autonomic function [106] suggesting a key role in the symptomatology noted in some patients. Alterations in autonomic tone also manifest themselves in chronic AF and the degree of dysfunction may be a method of risk stratification for SCD and HF [107].

The ECG provides a quick and readily accessible tool for the assessment of cardiac conduction and is of particular value in those with DM and DAN [108]. CAN may cause an increase in p-wave duration and dispersion and hence the ECG may be a useful tool for the prediction of AF [109].

## THE PRO-THROMBOTIC STATE: ADDED RISK OF THROMBOSIS? 

Individuals with AF are at significant risk of thromboembolic complications, most notably stroke. This risk is increased in the presence of co-existing obesity, MetS or DM, and yet with the exception of DM, they are not considered in current clinical guidelines for stroke risk stratification. The reported incidence of stroke in patients with DM and AF ranges between 3.6 and 8.6% per year [110]. The high incidence of thrombotic and thromboembolic events observed in these high risk groups may be attributable to a *pro-thrombotic state*. Indeed, obesity and DM predispose to thrombosis by adversely affecting all components of Virchow’s triad [111-113]. In relation to AF we will focus on hypercoagulability in DM and obesity, characterized by platelet activation, increased production and activation of clotting factors, hyperviscosity, and diminished fibrinolysis [114-116].

Obesity is associated with increased levels of several components of the coagulation cascade including tissue factor (TF), factors VII and VIII, von Willebrand factor (vWF), plasminogen activator inhibitor-1 (PAI-1), factor XIII B subunit and fibrinogen [117-120]. In addition, increased levels of protein C and prothrombin fragment 1 and 2 are present in obese subjects [121]. Body fat composition and distribution are also important, as plasma levels of clotting factors VII and X, proteins C, S and PAI-1 increase with both total and percentage central body fat [122]. Adipocytes produce TF which is essential for the generation of thrombin from prothrombin and TF activity is inhibited by a number of agents, including TF pathway inhibitor (TFPI) [123-125] whose activity is reduced in MetS and correlated with BMI [126]]. In contrast, the activity of pro-thrombotic factor VII increases with BMI [127]. The increased levels of TF and factor VII activate the coagulation protease cascade resulting in fibrin deposition and activation of platelets [128].

MetS is also associated with a pro-thrombotic state and individual components of this syndrome show independent relationships with clotting factors. Triglyceride levels are positively correlated with factor VII activity and PAI-1 levels are negatively correlated with adiponectin [127, 129]. The alteration in fibrinolysis associated with MetS is predominantly attributed to an increased serum PAI-1 concentration [130]. In the context of haemostasis, PAI-1 is the most significant adipokine and a two-fold increase in adipocyte PAI-1 mRNA levels is associated with a six-fold increase in its secretion from adipose tissue and a seven-fold increase in plasma PAI-1 activity in obesity [131]. However, its production in adipocytes appears to be fat depot-specific, in that visceral adipose tissue is a more significant contributor to plasma PAI-1 levels as compared with subcutaneous adipose tissue, and abdominal subcutaneous tissue secretes higher levels than femoral subcutaneous adipose tissue [132, 133].

In DM, circulating glucose exerts pro-thrombotic effects through several different mechanisms. Hyperglycaemia potentiates coagulation through elevated levels of thrombin-antithrombin complexes and soluble TF [134, 135]. Advanced glycation end products (AGE) induce TF mRNA expression and lead to increased TF levels [136, 137]. Factor VII levels also increase following induced-hyperglycaemia in both diabetic and non-diabetic subjects, and return to normal once euglycaemia is established [138]. Relative plasma viscosity (RPV) is also increased in diabetic subjects compared to healthy controls and increases progressively with worsening glycaemic control [139]. Insulin enhances PAI-1 production in adipocytes and hyperinsulinaemia results in elevated PAI-1 levels, thus attenuating the fibrinolytic pathway [134, 140].

Enhanced platelet activation is observed in subjects with MetS and DM through altered adipokine levels, increased thromboxane B(2) and activation of the platelet glycoprotein IIb/IIIa receptor [42, 141]. Leptin, an adipokine with predominantly nitric oxide-dependent vasodilator properties is elevated in obese subjects [142]. Platelets express the leptin receptor and the leptin-receptor complex results in promotion of platelet aggregation *via* synergistic action with adenosine diphosphate [143]. Adiponectin also plays a role in platelet aggregation, as knock-out animal models exhibit enhanced thrombus formation which is reversed by the delivery of adenovirus-mediated adiponectin [144]. Plasma adiponectin has been shown to be inversely correlated with platelet aggregation in patients with T2DM, HT or hypercholesterolaemia [145].

## INTERVENTIONS IN AF

### Stroke Risk in AF

The risk of thromboembolic complications and stroke in AF is non-linear. A number of risk factors have been identified that increase stroke risk in AF in a cumulative manner and have been incorporated into formal stroke risk stratification schemes to aid clinicians [110, 146, 147]. Over the past decade, these schemes have had a modest predictive value for stroke and thromboembolism, especially when patients are categorized into low, moderate, and high risk groups [147]. The main purpose for this risk stratification was to identify ‘high risk’ patients who might benefit from oral anticoagulant therapy in spite of its relative inconvenience. These risk categories however are essentially artificial divisions of a continuous spectrum of stroke risk, especially in the presence of multiple stroke risk factors [147, 148]. As such, the 2010 European Society of Cardiology (ESC) guidelines relaxed the emphasis on categorizing patients into low, moderate or high stroke risk by promoting an approach based on individual risk factors [148].

To complement the simple and extensively used CHADS_2_ (Congestive heart failure, Hypertension, Age >75 and Diabetes and Stroke/TIA) score, the ESC guidelines recommend the use of the newer CHA_2_DS_2_-VASc scheme [148, 149] emphasizing a more robust stroke risk assessment, and to help identify those who are truly ‘low risk’.

Unlike DM, obesity and MetS are not considered specific risk factors in such risk stratification schemes however there is some evidence to suggest they should. In a study examining the risk of left atrial thrombus in patients with AF, subjects with a BMI ≥ 27 had a markedly increased risk of left atrial appendage thrombus (OR 4.02 CI 95% 1.19-13.55, p=0.025) [150].

### Bleeding Risk

When making clinical decisions on suitability for anticoagulant therapy, clinicians need to carefully balance the benefit against the risk of bleeding. The latest ESC guidelines [148] provide a bleeding risk score (HAS-BLED), which is simpler than previously published schemes, only one of which has been derived and validated in an AF cohort [151]. Obesity and MetS have not been shown to influence bleeding risk.

### Antithrombotic Prophylaxis

Until recently, vitamin K antagonists (VKAs) such as warfarin were the only approved means of oral anticoagulant therapy for stroke prevention in AF, and ACC/AHA/ESC 2006/2010 guidelines recommend that patients with moderate-to-high risk of stroke should be considered for stroke prophylaxis with a VKA [152, 153].

The main drawback of VKA therapy is the requirement for regular monitoring of the INR in order to consistently achieve a dosage regimen which delivers a therapeutic range. The time within the therapeutic range is also a vital determinant for achieving effective protection against ischaemic stroke and minimising the risk of major haemorrhage. Good anticoagulation control, time in therapeutic range ≥70%, is associated with a low risk of stroke and bleeding events [154]. In addition, INR is affected by many factors including diet, drugs, alcohol and genetics which restrict the number of eligible patients who can take this therapy effectively [155].

The shortcomings of VKA have driven the development of oral antithrombotic agents that have a more predictable dose response in a broad range of patients and require less frequent, if any monitoring. The new oral non-VKA anticoagulant drugs can be divided into two categories: the oral factor Xa inhibitors and oral direct thrombin inhibitors, both of which on current data appear safer and more practical to use [156].

## RATE VS. RHYTHM CONTROL

### Rate Control

Rate-control treatment is based on pharmacological depression of conduction through the atrioventricular node. Three classes of drugs are commonly used for rate-control treatment: β-blockers, such as metoprolol or bisoprolol, non-dihydropyridine calcium antagonists (verapamil, dialtiazem) and digoxin. In the absence of pre-excitation, β-blockers are first-choice drugs to reduce the heart rate. Traditionally, there has been a reluctance to use β-blockers in patients with DM for fear of adverse effects on insulin resistance and an unawareness of hypoglycaemia. However, given the overall benefits of β-blockers in HF and CHD, conditions which are prevalent amongst diabetic patients, the recommendations regarding β-blockade remain the same. Clearly, intolerance in individuals should prompt switching to an alternative.

There is some emerging animal data which suggests that calcium channel blockers, such as a verapamil, may prevent loss of functional β-cell mass [157]. It has recently been identified that thioredoxin-interacting protein (TXNIP) may be a potential target to prevent loss of functional β-cell mass. Glucose and DM upregulate β-cell TXNIP expression, and TXNIP overexpression induces β-cell apoptosis. In contrast, genetic ablation of TXNIP promotes endogenous β-cell survival and prevents streptozotocin (STZ) and obesity-induced DM. Xu *et al*. have demonstrated that calcium channel blockers inhibit TXNIP expression in INS-1 cells and human islets. Also, orally administered verapamil reduced TXNIP expression and β-cell apoptosis, enhanced endogenous insulin levels, and rescued mice from STZ-induced DM. Furthermore, verapamil promoted β-cell survival and improved glucose homeostasis and insulin sensitivity in BTBR ob/ob mice. If human studies support these findings then calcium channel blockers may became the drug group of choice in diabetic patients without co-existing HF or CHD.

In patients who fail to respond to rate limiting drugs, non-pharmacological measures such as atrioventricular nodal ablation may be considered [158].

### Rhythm Control

Up to 50% of patients with recent onset AF revert back to sinus rhythm spontaneously [148]. If the patient does not revert spontaneously, they can be considered for either pharmacological or electrical cardioversion, especially for those who remain symptomatic despite adequate ventricular rate control. In patients with structural heart disease, such as CHD and systolic left ventricular dysfunction, class I antiarrhythmic drugs, including flecainide and propafenone, are contraindicated because of the potential increased proarrhythmia risk [148].

Amongst diabetic patients this can limit the options for drug therapy due to the higher incidence of HF and CHD. There is no data in the literature to indicate whether commonly used anti-arrhythmic medications to achieve cardioversion or maintain sinus rhythm are any less effective amongst diabetic or obese patients and therefore should be considered, bearing in mind the previously mentioned caveats. However, obesity has been implicated in a number of studies as a predictor of failed electrical cardioversion and earlier recurrence of AF [159-161]. By virtue of the high prevalence of obesity amongst diabetic patients, as high as 52% in T2DM in some cohorts [162], the more modest success rates in those with BMI >30 should be considered when deciding on management strategy. In those obese patients who fail external DC cardioversion, further options are: higher energy [163]; antero-posterior positioning of paddles [164]; fluoroscopy guided positioning of defibrillation paddles [165]; or internal DC cardioversion [166].

It should be emphasised that whatever strategy is chosen, the patient must be evaluated for long-term antithrombotic prophylaxis according to his/her risk profile.

### Patient-tailored Therapy

The decision to add rhythm control therapy to the management of AF requires an individual decision and should therefore be discussed at the beginning of AF management and revisited depending on response to treatment/side effects. Before choosing rate control alone as a long-term strategy, the physician should consider how permanent AF is likely to affect the individual patient in the future and how successful rhythm control is anticipated to be. AF related symptom burden is a key determinant in influencing the decision to opt for rate or rhythm control in addition to factors that may influence the likely success of a rhythm control strategy. These include a long history of AF, older age, more severe associated cardiovascular diseases, other associated medical conditions, and enlarged LA size.

The relatively low efficacy of prophylactic antiarrhythmic agents and the incidence of their potentially proarrhythmic effects has promoted the development of non-pharmacological strategies for prevention and control of AF, based on surgical or radiofrequency ablation.

### Atrial Catheter Ablation

The aim of catheter ablation for AF is to improve symptoms by eliminating the triggers or substrates that initiate and maintain the condition. In patients with paroxysmal AF, most triggers originate in or around the pulmonary veins with only about 10% being detected at the left atrial posterior wall, inter-atrial septum, coronary sinus, superior vena cava, and crista terminalis. As a result, electrical isolation of pulmonary veins alone with different energy sources is the cornerstone of the catheter ablation procedure for the treatment of paroxysmal AF, and can achieve clinical success in 64–71% of patients [167-169]. However, the clinical efficacy of catheter ablation for persistent AF is less favorable even with additional ablation approaches, including complex fractionated electrogram and multiple linear left atrial ablations to target atrial substrate (22–56%) [167-169]. Findings from a number of multi-centre prospective clinical trials [167-169], systematic reviews and meta-analyses [170-172] have consistently shown that catheter ablation is more effective than antiarrhythmic drug therapy for maintenance of sinus rhythm, especially in patients with paroxysmal AF who did not respond to initial treatment with antiarrhythmic drugs. Furthermore, successful catheter ablation of AF to maintain sinus rhythm was associated with improved symptoms and quality of life [173].

Nonetheless, catheter ablation is a complex interventional procedure that requires skilled operators and is not without risk. The use of three-dimensional electro-anatomical mapping systems, sometimes combined with robotic navigation, can provide a more accurate anatomical guide to target ablation in the atria. Furthermore, advances in catheter ablation technology—such as the circular and balloon ablation system and different energy sources, including bipolar and irrigated radiofrequency energy, cryo-ablation, microwave, and laser ablations—are promising techniques to try and improve the safety and efficacy of AF ablations. However, the best technique for catheter ablation is still unknown [174].

Catheter ablation is associated with a risk of major complications (about 3–4%), and several procedures are often needed to control recurrent AF or post-ablation atrial tachycardia [175]. Studies suggest that a substantial proportion of patients develop late recurrence of AF after catheter ablation [176]. Furthermore, there is no evidence to suggest that catheter ablation reduces stroke or mortality, beyond rhythm and symptom control. Whether catheter ablation can improve long-term clinical outcomes may be addressed in ongoing trials. Recurrences are often asymptomatic, and the proportion of asymptomatic paroxysms after ablation is increased [177]. As a result, clinical guidelines have recommended catheter ablation for patients with paroxysmal AF and minimal structural heart disease who remain symptomatic after initial anti-arrhythmic drug therapy [148]. In patients with structural heart disease or persistent AF, catheter ablation should be reserved for those who are refractory or intolerant to at least one antiarrhythmic drug or used as an alternative to amiodarone therapy.

### Catheter Ablation of AF in Patients with Diabetes

The increased prevalence of CHD in patients with T2DM often restricts the use of class Ia and Ic antiarrhythmic agents. As such, they may potentially glean a greater symptom benefit from ablation. There are now randomised data evaluating efficacy of catheter ablation in patients with T2DM [178-180]. From their study cohort of 263 patients Tang *et al*. compared those with T2DM (n=31) against those without (n=232) [180]. Although AF recurrence rates were not significantly different between the two groups, the trend suggested higher recurrence in patients with T2DM, 32.3% vs. 22.4% (p=0.24). This trend was to be expected given the important differences in clinical baseline characteristics: age (62.0 ± 10.8 vs. 56.1 ±10.6 years, P = 0.004), longer AF history (9.6 ± 9.3 vs. 6.7 ± 6.3 years, p=0.024), significantly larger left atrium size (41.1 ± 7.8 vs. 38.3 ± 5.8 mm, p=0.021), HT (58.1 vs. 35.8%, p=0.018) and structural heart disease (67.7 vs. 43.5%, p=0.011). Patients with DM also experienced a higher rate of complications, 29.0% vs. 8.2%, p=0.002). Forleo *et al*. randomized patients with T2DM to either radiofrequency ablation or anti-arrhythmic therapy [179]. Over a 12 month follow up period they found that AF recurrence rates were higher in the non-ablation arm (57.1% vs. 20%, p=0.001). Unlike Tang *et al*., complication rates were comparable to previous published data for left atrial catheter ablation.

A more recent study compared AF patients with abnormal glucose metabolism (T2DM or IGT) against those without [178]. They found that the substrates for AF differed significantly between the groups: Left atrial (108.4 ± 22.3 vs. 94.0 ± 17.5ms, p <0.001) total activation times were significantly longer in the patients with AF and an abnormal glucose metabolism compared to those normal glucose metabolism. Furthermore, left atrial (1.48±0.74 vs. 2.05±0.78mV, p<0.001) bipolar voltages were also significantly lower in those with AF and abnormal glucose metabolism. Not unexpectedly, these adverse substrate changes had an impact on AF recurrence rates. Over a follow up period of 18±6.4 months the AF recurrence rate was significantly greater in patients with abnormal glucose metabolism (18.5% vs. 8.0%, p<0.022). Complex fractionated atrial electrograms (CFAE) are well documented to be an important factor in the maintenance of AF in persistent AF. This has led to most electrophysiologists incorporating CFAE ablation as an integral component of ablation in addition to pulmonary vein isolation (PVI) and linear left atrial ablation. CFAE in persistent AF tend to occur in localised areas within the left atrium of slow conduction and low voltage amplitude signals thought to be related to atrial fibrosis [181]. Although there is no data in the literature reporting an increased burden of CFAE in diabetic patients, this is likely, given Chao *et al*.’s findings of significantly lower left atrial activation times and bipolar voltages in patients with DM/impaired glucose tolerance [178]. There may well be a case for adopting similar ablation strategies in diabetic patients with PAF, as are currently used in persistent AF cases, in an effort to reduce AF recurrence.

### Catheter Ablation of AF in Obese Patients

Success rates with catheter ablation in obese versus non-obese patients is comparable without a significant increase in complications. Mohanty *et al*. compared patients with a BMI of <25 and those ≥25 and demonstrated comparable freedom from AF at 12 months (69% vs. 63%, p=0.109, respectively) [162]. Interestingly, patients in the obese category reported a greater improvement in quality of life (QoL) scores compared to non-obese patients. This was, at least in part, due to their worse baseline QoL scores. In multivariable analysis BMI ≥25 and baseline QoL were independent predictors of QoL improvement. However, others have found that obesity and metabolic syndrome were independent predictors of late recurrence of AF[182].

Catheter ablation appears to be an effective treatment in patients with DM, obesity or both and they appear to have more to gain in terms of symptomatic improvement. However, although there is potentially a greater symptom benefit to be gained by obese patients from catheter ablation, and small studies demonstrating comparable procedural risk, in practice, most electrophysiologists will choose not to ablate very obese patients. Catheter ablation in these patients can prove very challenging; venous access is frequently difficult, fluoroscopic imaging is often suboptimal and even sedation/general anaesthesia proves much more challenging.

### Upstream Therapies

Increasing evidence has shown that AF development and perpetuation depend on both electrophysiological and structural remodeling of the atrium [183]. Inflammation and oxidative stress have been linked to atrial remodeling and have also been implicated with AF recurrence after catheter ablation [184-186]. The potential beneficial effects of anti-inflammatory and antioxidant drugs such as statins, RAAS inhibitors and corticosteroids, on AF recurrence after catheter ablation has attracted much interest over the last 5 years [185-188]. Unfortunately, their potential has remained unfulfilled; angiotensin-converting enzyme inhibitors, ARBs and statins have failed to show any reduction in AF recurrence [189-191]. Omega-3 polyunsaturated fatty acid supplementation has not been definitively shown to have a positive impact on incidence or recurrence of AF. In fact, the weight of evidence is mostly against any benefit [192-195].

A recent 150 patient prospective observational study examined the effect of pioglitazone on the outcome of catheter ablation in patients with paroxysmal AF and T2DM [196]. Consecutive patients undergoing catheter ablation were divided into those who received pioglitazone before ablation or not. 51 patients treated with pioglitazone and 99 control subjects were followed up for at least 15 months after ablation. After a single ablation, sinus rhythm was maintained in 44 patients (86.3%) in the pioglitazone group vs. 70 patients (70.7%) in the control group (p=0.034), without antiarrhythmic drug during a mean follow-up of 22.9 ± 5.1 months. The second ablation was performed in 5 patients (9.8%) from the pioglitazone group and in 24 patients (24.2%) from the control group (p=0.034). Pioglitazone is a peroxisome proliferator activated receptor-gamma (PPARγ) agonist and represents a class of anti-diabetic agents that reduce insulin resistance mainly by activating PPARγ. Accumulating evidence suggests that PPARγ agonists exert modulatory effects on growth factor release, cell proliferation and migration, extracellular matrix remodeling, and cell cycle progression and differentiation [197]. Experimental evidence demonstrates that PPARγ agonists inhibit macrophage activation and the associated inflammatory cytokines [198] as well as suppress superoxide production and induce antioxidant enzymes [183]. Additionally, clinical studies suggest that PPARγ agonists reduce C-reactive protein (CRP) levels in diabetic and non-diabetic patients, independently of glycaemic control [199]. But of course it is important to remember that these drugs are contraindicated in NYHA class III and IV HF and therefore in the context of patients with AF and HF will have limited use. Furthermore because PPARγ agonists also cause weight gain which can increased AF risk the use of this class of drugs seems counterintuitive.

Bariatric surgery is the most reliable way of achieving and maintaining significant weight loss in morbidly obese individuals and is being performed in rapidly growing numbers across the globe [200, 201]. Weight loss secondary to bariatric surgery or lifestyle modification may reverse the hypercoagulable state in obesity by a reduction in thrombin generation and PAI-1 and TF levels [52], [202]. Clearly, there is a need for additional studies to further establish the most cost-effective interventions to reduce the pro-thrombotic state and stroke risk.

## CONCLUSION

Obesity, MetS and DM through a complex interplay of metabolic, inflammatory and neural mechanisms, create an environment conducive to AF and pose unique challenges in the treatment of this common arrhythmia. In addition, the presence of a pro-thrombotic state requires targeted therapeutic interventions if we are to avoid an epidemic of AF and associated stroke. Current evidence challenges the traditional paradigm which excludes obesity for risk stratification in AF.
